# Retracted: Exploring Drug Targets in Isoprenoid Biosynthetic Pathway for *Plasmodium falciparum*

**DOI:** 10.1155/2022/8426183

**Published:** 2022-03-15

**Authors:** Biochemistry Research International

*Biochemistry Research International* has retracted the article titled “Exploring Drug Targets in Isoprenoid Biosynthetic Pathway for *Plasmodium falciparum*” [1]. The article was found to contain a substantial amount of material from previously published articles [2–6], including the following sources:

- Baichen Zhang, Kristin M. Watts, Dana Hodge, Lisa M. Kemp, David A. Hunstad, Leslie M. Hicks, Audrey R. Odom. “A Second Target of the Antimalarial and Antibacterial Agent Fosmidomycin Revealed by Cellular Metabolic Profiling”, Biochemistry, 2011. https://doi.org/10.1021/bi200113y. (Cited as reference 3)
- Cyrille Y. Botté, Faustine Dubar, Geoffrey I. McFadden, Eric Maréchal, Christophe Biot. “Plasmodium falciparum Apicoplast Drugs: Targets or Off-Targets?”, Chemical Reviews, 2011. https://doi.org/10.1021/cr200258w. (Not cited)
- J. Kipchirchir Bitok, Caren Freel Meyers. “2-Methyl- -erythritol 4-Phosphate Enhances and Sustains Cyclodiphosphate Synthase IspF Activity”, ACS Chemical Biology, 2012. https://doi.org/10.1021/cb300243w. (Not cited)
- Fabiana Morandi Jordão, Emília Akemi Kimura, Alejandro Miguel Katzin. “Isoprenoid biosynthesis in the erythrocytic stages of Plasmodium falciparum”, Memórias do Instituto Oswaldo Cruz, 2011. https://doi.org/10.1590/S0074-02762011000900018. (Cited as reference 6)
- Lisheng Deng, Jiasheng Diao, Pinhong Chen, Venugopal Pujari et al. “Inhibition of 1-Deoxy-D-Xylulose-5-Phosphate Reductoisomerase by Lipophilic Phosphonates: SAR, QSAR, and Crystallographic Studies”, Journal of Medicinal Chemistry, 2011. https://doi.org/10.1021/jm200363d. (Not cited)

The authors disagree with this retraction.
